# Incontinentia Pigmenti; a Rare Multisystem Disorder: Case Report of a 10-Year-Old Girl

**Published:** 2016-09

**Authors:** Rezvan Rafatjoo, Amene Taghdisi Kashani

**Affiliations:** 1 Dept. of Pediatric Dentistry, School of Dentistry, Hamedan University of Medical Sciences, Hamedan, Iran.; 2 Dept. of Pediatric Dentistry, School of Dentistry, Kashan University of Medical Sciences, Kashan, Iran.

**Keywords:** Genetic Diseases, X-Linked, Incontinentia Pigmenti, Dental Anomalies

## Abstract

Incontinentia pigmenti is a rare genodermatosis in which the skin involvement occurs in all patients. Additionally, other ectodermal tissues may be affected such as the central nervous system, eyes, hair, nails and teeth. The disease has an X-linked dominant inheritance pattern. But in our case, there was a mutation in the body cells due to incontinentia pigmenti. The dermatological findings occur in four successive phases. We report the case of a 10-year-old female presented cutaneous, dental and ophthalmic characteristic with 3 years follow-up. Dental anomalies such as hypodontia, peg-shaped anterior teeth, malformed primary and permanent teeth, and delayed eruption were seen in our patient.

## Introduction

Incontinentia Pigmenti (IP), also known as Bloch-Sulzberger syndrome, is a rare X-linked dominant genodermatosis. It is a multisystem, ectodermal and mesodermal disorder accompanied by dermatologic, dental and ocular features. In a minority of cases, it may be associated with neurologic deficit.[1-4]

The mutation of NEMO (NF-kappa-B essential modulator) also known as IKK-γ/IKBKG (inhibitor of nuclear factor kappa-B kinase subunit gamma) gene which is located on chromosome Xq28, is believed to play a role in the pathogenesis of this disease.[5] NEMO/IKK-γ helps activate NF-κB, which controls the expression of multiple genes, including cytokines and chemokines, and protects cells against apoptosis.[6] A lack of NEMO/IKK-γ, therefore, causes a lack of active NF-κB, which makes cells more prone to apoptosis. This report describes a case of IP in a girl with dermatologic, orodental, and eyes signs, as well as skin lesions. The estimated prevalence of IP was stated to be 0.2 in 100,000 based on the data of 386 diagnosed IP patients who were reported in the available literature published during the period of 2000–2013.[7] In more than 80% of patients, dental anomalies represent the most common non-cutaneous manifestation of IP. Hypodontia, microdontia (as pegged and conically-shape teeth), delayed eruption and accessory cusps are frequently reported, and both primary and permanent dentitions may be affected. The computed tomography (CT) of the brain showed evidence of cerebral infarct which is consistent with the diagnosis of IP. The early diagnosis of IP and subsequent assessment of medical factors can impact on the long-term prognosis of each case. In the absence of ophthalmic and central nervous system involvement, the onus monitors and manages the cutaneous and dental manifestations if required. The significant absence and malformation of teeth can affect proper facial development, mastication, speech development, appearance and self-esteem in the growing child.[7] Parents should be made aware of the common occurrence of late eruption, missing teeth, and the effects on the developing dentition. It is also important to ensure the nutritional needs are being met.[8] The practice of thorough oral hygiene methods and healthy eating habits should be encouraged to help preserve the existing dentition for as long as possible.

## Case Report

A 10-year-old girl referred to a dentist for abnormal dentition. She was born of a 40-year-old mother, at full term after an uneventful pregnancy.

**Figure 1:**
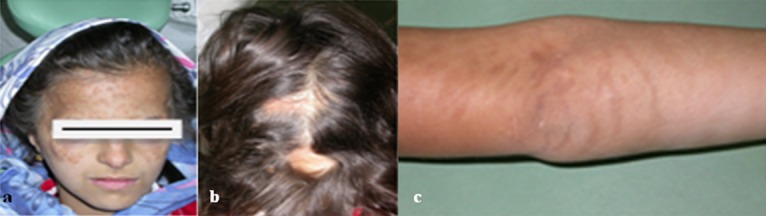
a: Hyperpigmentation on the face, b: Clinical view of the hair with alopecia,  c: Hyperpigmented lesions on the hand

Her skin had been fiery red at the birth time and vesicles had developed shortly afterwards. They had been replaced by verrucous lesions after a few weeks. Then the lesions had cleared gradually and left linear pigmentation. On clinical examination, hyperpigmented lines were seen in her face and limbs. (Figure 1)

**Figure 2:**
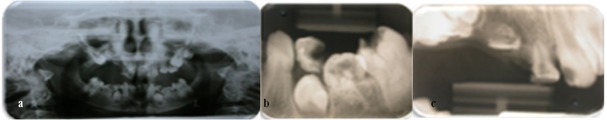
a: Panoramic radiography, b: Left mandibular parallel radiography, c: Right maxillary parallel radiography

Hypopigmented atrophic streaks were also seen on her upper limbs. History of similar disease was not present in her family. The disease was diagnosed as IP. Her genetic counseling was otherwise normal. Intra- and extra- oral examinations, including panoramic and periapical radiographs were completed. (Figure 2) The soft tissue was normal and only 10 primary teeth were present.

**Figure 3:**
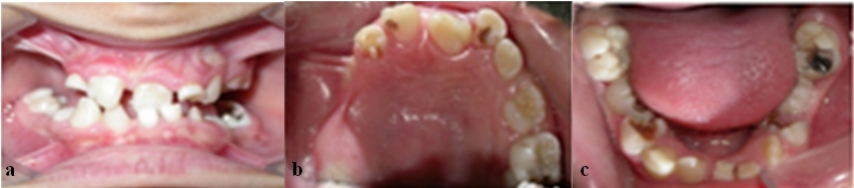
a: The patient’s clinical view in centric occlusion,  b: Clinical view of the maxilla, c: Clinical view of the mandible

In the oral examination of the patient, some of both mandibular and maxillary anterior teeth were conical and in the radiograph, the primary teeth roots were abnormal. The first right maxillary permanent molars showed a delayed development compared to their mandibular counterparts. The teeth had abnormal root morphology. Multiple carious lesions were seen in the primary and permanent mandibular molars and in the maxillary permanent central incisors. The interdental spaces were visible due to some missed teeth. There was sub-mucosal cleft palate and the conical condition of some other teeth. No hypoplasia or hypo calcification was seen on her teeth. Radiographs revealed 13 permanent teeth, including 3 mandibular anterior teeth, (# 33, #42-43) the first right premolar (# 44) and molar, (#46) first left premolar (#34) and molar, (#36) three maxillary incisors and first left and right and second left molar. (#16, #11, #21-22, #26-27) Mandibular primary second molars and right maxillary primary first and second molars were ankylosed. The first permanent molars in maxilla had ectopic eruption. (Figure 3)

## Discussion

IP is inherited in an X-linked dominant manner. Therefore, more than 95% of patients are female infants.[9] In males, it is usually lethal and most of the affected male fetuses result in miscarriage or stillbirth.[4] In our case, according to her genetic counseling, the IP was a result of a mutation in some of the body cells and there was no cause of X-linked dominant inheritance pattern. IP is hereditary in 10%-25% of cases.[10]

Dermatologic findings are often the first observed signs of IP and are present in nearly all patients.[7] In most cases, the first of skin changes appear before 6 weeks of age.[4] Progressive cutaneous manifestations are the main clinical feature of the disease and classically evolve through four stages. However, their sequence may be irregular and some of stages may overlap with others or not appear at all. Stage 1 (vesicular stage) is presented at birth or within the first two weeks in 90% of patients and is characterized by a rash of erythematous blisters, which often appear along the lines of Blaschko. Biopsy characteristically exhibits spongiotic dermatitis with massive intra-epidermal and dermal eosinophilia.[7] Stage 2 (verrucous stage) occurs in about 70% of patients. Eruption of hyperkeratotic verrucous papules and plaques develop over the healing blisters. It usually appears within 2 months and disappears within six months. Hyperkeratosis, dyskeratosis, acanthosis and papillomatosis are present in this stage.[11-12] Stage 3 (hyperpigmented stage) is classically the hallmark of IP. Nearly 98% of patients experience stage 3. The pigmentations range from blue-grey or slate to brown, and occur in streaks or whorls. It generally develops within the first few months of life and tends to fade by adolescence. Melanophages in the dermis and vacuolization of basal cells are the most common findings.[4, 13] Stage 4 (atrophic/hypopigmented stage) occurs in adolescence and persists into adulthood.

Pale, hairless patches or streaks, and scar-like lesions are mostly found on lower legs. Such changes are mostly permanent and often the only signs of skin involvement in adult patients. It presents as atrophy and thinning of the epidermis with the absence of skin appendages.[4, 14-16] The vesicular stage is most often observed at birth or within the first two weeks of life in IP, which coincides with our case. The patient presented with stage 1 and gradually developed to stages 2 and 3. According to the follow-up examinations, we believe that current clinical presentation of patients can considered as hyper pigmentation stage. Since the patient was 10-year-old, she was in stage 4 and scar-like lesions were mostly found on her face, upper limbs, trunk, and neck. Cutaneous lesions may also be accompanied by defects of cutaneous appendages in the form of vertex alopecia, ridged, pitted, or dystrophic nails.[17] Extracutaneous manifestations occur in various ways in about 70-80% of IP patients. Dental abnormalities are the most common types and affect more than 80% of patients with delayed dentition, partial anodontia, conical or peg-shaped teeth, or absence of teeth. It is reported that 30-50% of patients exhibit neurologic deficiency identified as seizures, mental retardation, developmental delays, spastic paralysis, ataxia and motor dysfunction.[18-20] Ocular abnormalities are also observed in about 30% of patients including strabismus, cataracts, optic atrophy, retinal dysfunction, uveitis, nystagmus, and blindness. Additionally, there are occasional reports on skeletal and structural anomalies such as somatic asymmetry, skull deformities, spina bifida, dwarfism, syndactyly, extra ribs, primary pulmonary hypertension, and cardiopulmonary failure. Keratotic tumors in late adolescence may involute spontaneously. Several cases of IP have been associated with cancer in childhood.[21-22] Clinical findings associated with physical characteristics are enough to define the diagnosis. Recently, diagnostic criteria were proposed for the syndrome. The typical cutaneous alterations that could be one of the evolutionary phases of the disease were the major criteria. The minor criteria of this disease could be dental, ocular, neurological, osseous and immunological alterations. In our case, there were major (scar-like lesions, alopecia) and some minor criteria (dental, ocular). The cutaneous manifestations do not need specific treatment. Spontaneous resolution of the lesions occurs normally. Emollients and topical corticoids can be used in the first stages of the disease. Secondary bacterial infections may occur and must be treated with antibiotics.[22-23]

The extracutaneous manifestation must be managed by a multidisciplinary team and this management varies according to the affected organ. Furthermore, genetic counseling is also important since the disorder is of autosomal dominant transmission. Therefore, in spite of the rarity of this pathology, one must be alert for early diagnosis, recognizing the typical cutaneous manifestations of each evolutionary phase of the disease, adequate genetic counseling and better management of extracutaneous manifestations when these are present.[23]

In our case, a 10-year-old girl referred to the Department of Pediatric Dentistry. Gray-brown hyperpigmentations were observed on her face and skin of the arms, neck and trunk. The patient’s mother declared   that former stages of skin lesion have been occurred early after birth. Multiple areas of alopecia were seen on the scalp and eyebrow region. Minor eye abnormalities were seen in form of euryblepharon. The oral findings included hypodontia, notched central incisors, malformed first molars with multiple cusps such as mulberry molar, caries and sensitivity to stimulants, anterior crossbite, over-retained primary teeth and disrupted eruption sequence. The patient had normal intellectual ability and no other signs such as neurologic deficiency, identified as seizures, mental retardation, developmental delays, spastic paralysis, ataxia and motor dysfunction were present. The patient underwent dental treatment as needed, her teeth were restored, all carious lesions were removed, and oral hygiene improved. Further treatment was postponed until complete eruption of the remained teeth. The growth of bone and gingiva make the orthodontic and prosthetic treatment possible. 

## Conclusion

Long-term and close cooperation between dermatologists, pediatricians, neurologists, genetic counselors, and even dentists is crucial for better understanding of IP and prediction of the occurrence of the potential anomalies that may later occur in life. 
